# Deciphering Platelet Transcriptomic Profiles in Antiphospholipid Syndrome: Insights into Their Role as Immunological Players

**DOI:** 10.3390/ijms27125428

**Published:** 2026-06-16

**Authors:** Yaqing Yang, Haiyue Jiang, Zihan Tang, Honglei Liu, Xiaobing Cheng, Yutong Su, Junna Ye, Qiongyi Hu, Yue Sun, Jianfen Meng, Huihui Chi, Zhuochao Zhou, Jinchao Jia, Mengyan Wang, Yuning Ma, Hui Shi, Jialin Teng, Chengde Yang, Tingting Liu

**Affiliations:** Department of Rheumatology and Immunology, Ruijin Hospital, Shanghai Jiao Tong University School of Medicine, Shanghai 200025, China

**Keywords:** antiphospholipid syndrome, platelet transcriptomics, immunothrombosis, toll-like receptors, machine learning, biomarkers

## Abstract

Antiphospholipid syndrome (APS) is a systemic autoimmune disorder driven by antiphospholipid antibodies (aPLs), primarily characterized by recurrent thrombosis and pregnancy morbidity. Transcriptomic analyses of other immune compartments have provided insights into APS pathogenesis. However, despite the central role of platelets in APS pathophysiology, their transcriptomic features remain largely unexplored. We therefore aimed to characterize the platelet transcriptomic landscape in APS and identify key immune-thrombotic pathways. RNA sequencing and bioinformatic analyses of platelets from 43 APS patients, 20 asymptomatic aPL carriers, and 20 healthy controls revealed distinct proinflammatory transcriptional reprogramming, consistent with platelets as active thrombo-inflammatory hubs. APS platelets exhibited marked activation of innate immune (Toll-like and NOD-like receptors) and neutrophil pathways, which may contribute to disease propagation through a functional platelet–neutrophil axis. A 15-gene classifier (e.g., *FCER1G*, *MAP4K4*) discriminated APS from controls with high accuracy (Area Under the Curve 0.901–0.948). The classifier also highlighted molecular differences between APS and asymptomatic aPL carriers relevant to disease progression. Network analysis suggested that anti-β2-glycoprotein I antibodies were associated with unique coagulation–immunity modules, while arterial and venous thrombosis displayed divergent cytoskeletal-energetic and lipid-signaling signatures, respectively. Together, these findings suggest that platelets in APS acquire a distinct proinflammatory phenotype and function as key immunothrombotic interfaces, supporting disease stratification and platelet-derived diagnostic strategies.

## 1. Introduction

Antiphospholipid syndrome (APS) is a systemic autoimmune disorder driven by antiphospholipid antibodies (aPLs). It is primarily characterized by recurrent thrombosis and pregnancy morbidity. In addition, it may present with thrombocytopenia, cardiac valve abnormalities, livedo reticularis, and other systemic manifestations [1]. aPLs are a heterogeneous group of antibodies that bind to phospholipids and phospholipid-binding proteins. Lupus anticoagulant (LAC), anticardiolipin (aCL) antibodies, and anti-β2-glycoprotein I (aβ2GPI) antibodies are representative aPLs and included in the 2023 APS classification criteria [2]. APLs play a central role in the pathogenesis of APS by inducing the activation of endothelial cells, platelets, and leukocytes. This cellular activation fosters a prothrombotic intravascular milieu and facilitates the initiation of autoimmune responses and downstream inflammatory signaling cascades [3].

The “two-hit hypothesis” provides a conceptual framework for understanding the pathogenesis of APS. According to this model, the presence of aPLs constitutes the first hit, priming endothelial cells, platelets, and immune cells toward a prothrombotic and proinflammatory state. A subsequent triggering event—the second hit—such as infection, trauma, or inflammation, precipitates overt thrombotic manifestations or pregnancy-related complications [4]. Within this milieu, platelets adopt an aPL-educated phenotype marked not only by heightened prothrombotic responsiveness but also by enhanced capacity to regulate immune activation.

Platelets are a critical component of the hemostasis system and have been extensively studied in this context. In contrast, their immunological functions have received comparatively less attention. Accumulating evidence from previous studies suggests that platelets play an active role in both host defense against pathogens and sterile inflammation by interacting with components of the innate and adaptive immune systems. They express multiple pattern recognition receptors (PRRs), enabling them to detect pathogen-associated and damage-associated molecular patterns. Moreover, platelets actively engage in immune modulation through interactions with the complement system [5], neutrophils [6] and monocytes [7], thereby contributing to inflammatory responses and host defense. In addition, platelets contribute to adaptive immune regulation by influencing antigen-presenting cell (APC) maturation [8], promoting T-cell activation and differentiation [9].

Multiple studies have demonstrated that aPLs can activate platelets and promote thrombosis [10,11]. Our previous research revealed that anti-β2GPI antibodies contribute to thrombosis by activating platelets through the mammalian target of rapamycin complex 2 (mTORC2) signaling pathway [12,13]. More specifically, aPLs, particularly anti-β2GPI antibodies, can directly bind to platelet surface receptors, inducing activation, aggregation, and pro-inflammatory signaling [14]. Activated platelets also induce enhanced complement fixation [15], increase microparticle release, and also release more platelet-derived chemokines (e.g., platelet factor 4) [16]. These findings suggest that in APS, platelets not only participate in thrombosis but also contribute to immune dysregulation, amplifying inflammatory and autoimmune responses.

Platelets are equipped with pre-formed mRNA, proteins, and bioactive molecules, enabling them to rapidly sense and respond to external stimuli [17]. Increasing evidence suggests that the transcriptional landscape of platelets is closely linked to their functional state, and deciphering these characteristics may offer critical insights into platelet-mediated mechanisms in disease [18]. In APS, transcriptomic analyses of other immune compartments have provided valuable information on disease activity and pathogenesis, for example, overexpression of P-selectin glycoprotein ligand-1 in neutrophils [19], and interferon-regulated genes as key drivers of transcriptomic alterations in whole blood from patients with primary thrombotic APS [20]. Moreover, a large-cohort RNA-seq analysis in systemic lupus erythematosus (SLE) demonstrated that SLE-APS is associated with a dampened interferon signature and that venous and arterial thrombotic events are characterized by distinct gene expression patterns [21]. These findings highlight the value of transcriptomic profiling in elucidating disease mechanisms in APS. However, despite the central role of platelets in APS pathophysiology, their transcriptomic features remain largely unexplored.

To address this gap, we performed RNA sequencing of platelets from 63 aPL-positive individuals (including 43 APS patients and 20 aPL carriers) and 20 healthy controls (HCs), integrating transcriptomic profiles with clinical phenotypes and antibody characteristics. Using an integrative approach combining machine learning, protein–protein interaction (PPI) network analysis, gene set enrichment analysis (GSEA), and weighted gene co-expression network analysis (WGCNA)—we aimed to delineate the platelet transcriptional landscape in APS, identify key immune–thrombotic pathways, and evaluate platelet-derived mRNA signatures as candidate diagnostic biomarkers. These findings offer new insights into the immunothrombotic role of platelets in APS and provide a foundation for novel diagnostic and therapeutic strategies.

## 2. Results

### 2.1. Patient Characteristics

We enrolled 43 patients with APS and 20 asymptomatic aPL carriers from the APS-SH cohort at Ruijin Hospital, along with 20 age- and sex-matched healthy controls (Table 1). Among the APS patients, 25 had venous thrombosis, 21 had arterial thrombosis, 4 had microvascular thrombosis, 8 experienced obstetric morbidity, 4 had cardiac valve disease and 11 presented with thrombocytopenia. Based on the 2023 American College of Rheumatology (ACR)/European League Against Rheumatism (EULAR) criteria, high-titer aPLs were detected in 22 APS patients (51.1%) and 14 aPL carriers (70%). Triple-positive aPL profiles were observed in 17 APS patients (39.5%) and 11 aPL carriers (55%).

### 2.2. Integrative Transcriptomic Analysis of Platelets in aPL-Positive Individuals with Healthy Controls and Public Datasets

The transcriptome of aPL-positive individuals was examined, and Linear Discriminant Analysis (LDA) showed distinct separation among APS patients, aPL carriers, and HCs, indicating substantial transcriptomic divergence (Supplementary Figure S1A). Differential expression analysis using limma with empirical Bayes moderation identified 1842 genes with significant variation across the three groups (*p* < 0.05, omnibus F-test). Pairwise comparisons (|log_2_ Fold Change (log_2_FC)| ≥ 1, *p* < 0.05) revealed 1199, 821, and 462 differentially expressed genes (DEGs) in APS vs. HC, aPL carriers vs. HC, and APS vs. aPL carriers, respectively, partially visualized in the heatmap (Supplementary Figure S1B).

**Figure 1:**
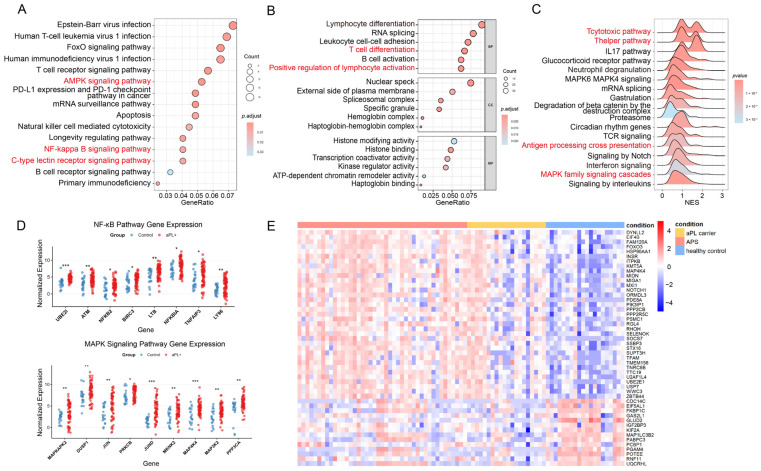
Platelet transcriptomic signature in aPL-positive patients (APS and aPL carrier) versus healthy controls: platelet activation as a central feature. (**A**) Dot plot showing KEGG analysis. (**B**) Dot plot showing GO analysis. (**C**) Ridge plot showing the GSEA of canonical pathways. (**D**) Dot plot showing key genes in NF-κB pathway and MAPK signaling pathway. (**E**) Heatmap showing the expression profile of the 49 representative genes found as DEGs between APS, aPL carriers and HC. Expression values were z-score normalized. Top annotation row shows the condition of each sample, colored salmon for patients with APS, yellow for aPL carriers and blue for HC. aPL, antiphospholipid antibodies; HC, healthy controls; APS, antiphospholipid syndrome; DEGs, differentially expressed genes; KEGG, Kyoto Encyclopedia of Genes and Genomes; GO, gene ontology; GSEA, gene set enrichment analysis; NES, normalized enrichment score. * *p* < 0.05; ** *p* < 0.01; *** *p* < 0.001. Red-colored elements in this figure are those specifically highlighted and discussed in the main text.

To identify the core transcriptional perturbations common to the entire aPL-positive spectrum, we compared all aPL-positive individuals (APS patients + aPL carriers) against HCs. Pathway enrichment analysis indicated distinct but overlapping molecular signatures. Kyoto Encyclopedia of Genes and Genomes (KEGG) analysis showed significant enrichment of C-type lectin receptor signaling, AMP-activated protein kinase (AMPK) signaling, and Nuclear Factor Kappa B (NF-κB) signaling pathways, pointing to both immune dysregulation and altered metabolic states (Figure 1A). Concurrently, Gene Ontology (GO) terms related to adaptive immunity—including lymphocyte activation and T-cell differentiation—were markedly enriched (Figure 1B), while canonical pathway GSEA further implicated platelets in platelet-T-cell interactions, Mitogen-Activated Protein Kinase (MAPK) cascades, and antigen processing (Figure 1C). Key genes involved in the NF-κB and MAPK pathways are shown in a dot plot (Figure 1D). These findings are consistent with a state of heightened platelet activation and immune dysregulation in aPL-positive patients.

Compared with HC, aPL-positive patients exhibited 462 up-regulated and 448 down-regulated DEGs, partially represented in the heatmap (Figure 1E). To assess specificity, we compared our APS platelet transcriptome with published datasets from SLE and sepsis [22,23]. Although 13/43 patients (30.2%) had secondary APS associated with SLE, the transcriptional overlap was limited: 109/592 APS-upregulated DEGs (18.4%) and 46/607 downregulated DEGs (7.6%) were co-regulated in SLE, suggesting partial convergence reflecting shared immune–inflammatory mechanisms. In contrast, the overlap with sepsis was markedly lower, with only 35 co-directional DEGs shared (Supplementary Figure S2A). This divergence supports disease-specific platelet programs in APS distinct from general inflammation.

To evaluate the potential contribution of platelet age to the APS-associated transcriptome, APS-related DEGs were compared with a published reticulated platelet transcriptome [24]. Among 1199 co-directional APS-associated DEGs, 67 genes (5.6%) overlapped with those identified in reticulated platelets (Supplementary Figure S2B), indicating a limited degree of transcriptomic overlap. Collectively, these comparative analyses suggest that the platelet transcriptome in APS is both disease-specific and intrinsic, suggesting a stable reprogramming of platelet biology in APS pathophysiology.

### 2.3. Machine Learning Nominates Candidate Transcriptional Biomarkers Distinguishing APS from HCs

Differential expression analysis between APS and HC identified 592 up-regulated and 607 down-regulated DEGs. Further pathway analysis suggested platelet involvement in immune regulation in APS. Specifically, platelet-enriched pathways included those related to PRRs, lymphocyte differentiation, neutrophil degranulation, antigen processing cross presentation and interferon signaling (Supplementary Figure S3).

**Figure 2:**
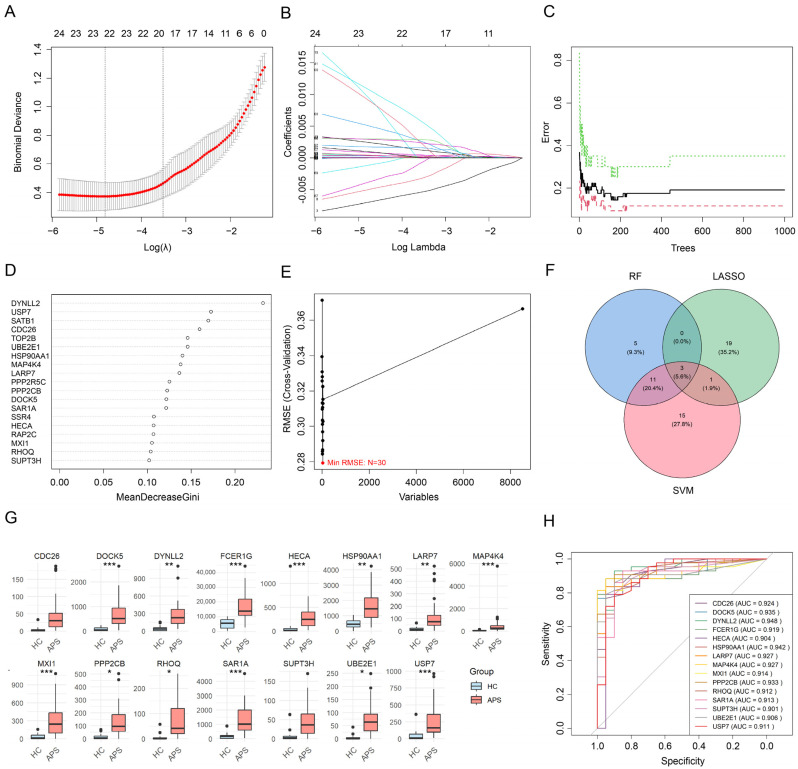
Machine learning identifies key discriminative genes in APS versus HCs. (**A**,**B**) The performance of 10-fold cross verification for tuning parameter in selection LASSO. (**C**) Random Forest error rate versus the number of classification trees. (**D**) Top 19 relatively important genes selected by Random Forest. (**E**) Recursive feature elimination (SVM–RFE) algorithm selection. (**F**) Venn plot showing the number and percentage of key genes identified by three machine learning methods. Intersections denote genes screened by two or three methods. (**G**) Boxplot of key discriminative gene expression in APS patients and HC. (**H**) ROC curves and AUC rate of the feature genes. HC, healthy controls; APS, antiphospholipid syndrome; ROC, Receiver Operating Characteristic; AUC, Area Under the Curve. * *p* < 0.05; ** *p* < 0.01; *** *p* < 0.001.

To further identify robust biomarkers for APS, we applied three supervised machine learning algorithms: Least Absolute Shrinkage and Selection Operator (LASSO) regression, Random Forest (RF), and Support Vector Machine-Recursive Feature Elimination (SVM-RFE). LASSO selected 23 candidate genes based on the optimal regularization parameter λ determined via cross-validation (Figure 2A,B). RF ranked feature importance using the Mean Decrease Gini index and retained the top 19 genes (Figure 2C,D), while SVM-RFE identified 30 genes corresponding to the minimum cross-validation error (Figure 2E). To enhance reliability and reduce method-specific bias, genes identified by at least two algorithms were considered key candidates, yielding 15 overlapping genes: *CDC26*, *DOCK5*, *DYNLL2*, *FCER1G*, *HECA*, *HSP90AA1*, *LARP7*, *MAP4K4*, *MXI1*, *PPP2CB*, *RHOQ*, *SAR1A*, *SUPT3H*, *UBE2E1*, and *USP7* (Figure 2F,G). Subsequent Receiver Operating Characteristic (ROC) analysis showed strong discriminative performance for these genes, with Area Under the Curve (AUC) values ranging from 0.901 to 0.948 (Figure 2H), suggesting their strong discriminative capacity in our cohort.

Among these, six genes are implicated in immune signaling and inflammation. *DOCK5* participates in RHO GTPase cycling, regulating chemotaxis and ROS production [25,26]. *FCER1G* encodes the Fc receptor γ-chain (FcRγ), an immunoreceptor tyrosine-based activation motif (ITAM)-containing adaptor mediating downstream signaling and antigen-presenting cell maturation [27,28]. *HSP90AA1*, a molecular chaperone, contributes to stress responses and associates with NOD-like receptor signaling [29,30]. *MAP4K4* amplifies immune responses via MAPK and NF-κB pathways, engaging in a TNF-α feedback loop driving chronic inflammation [31,32,33]. *SAR1A* facilitates STING trafficking and subsequent interferon pathway activation [34,35]. *USP7* promotes SLE activity by stabilizing the IFNα receptor [36,37].

Taken together, these findings suggest candidate transcriptional features that distinguish APS patients from healthy controls. The strong discriminative performance (AUC 0.901–0.948) in our cohort highlights their potential for further development as diagnostic biomarkers, pending validation in independent clinical samples.

### 2.4. Transcriptomic Signatures Implicate Toll-like Receptor Activation in APS Thrombosis

Platelets from aPL carriers showed enrichment of pathways related to viral infection, complement activation, lymphocyte differentiation and cytokine signaling compared with healthy controls (Supplementary Figure S4), yet long-term follow-up revealed no thrombotic events, prompting investigation into mechanisms distinguishing carriers from APS patients.

**Figure 3:**
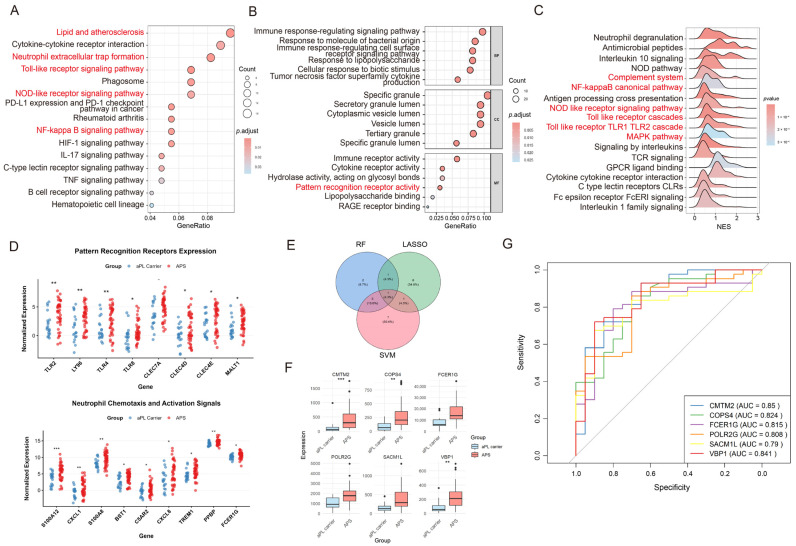
Platelet transcriptomic signature in APS versus aPL carriers: enhanced Toll-like receptor signaling in APS. (**A**) Dot plot showing KEGG analysis. (**B**) Dot plot showing GO analysis. (**C**) Ridge plot showing the GSEA of canonical pathways. (**D**) Dot plot showing key genes in pattern recognition receptor pathways and neutrophil chemotaxis and activation signals. (**E**) Venn plot showing the number and percentage of key genes identified by three machine learning methods. Intersections denote genes screened by two or three methods. (**F**) Boxplot of key discriminative gene expression in APS patients and aPL carriers. (**G**) ROC curves and AUC rate of the feature genes. APS, antiphospholipid syndrome; aPL, antiphospholipid antibodies; KEGG, Kyoto Encyclopedia of Genes and Genomes; GO, gene ontology; GSEA, gene set enrichment analysis; ROC, Receiver Operating Characteristic; AUC, Area Under the Curve. * *p* < 0.05; ** *p* < 0.01; *** *p* < 0.001. Red-colored elements in this figure are those specifically highlighted and discussed in the main text.

Compared with aPL carriers, APS patients displayed 255 up-regulated and 207 down-regulated genes, with pathway analyses revealing marked activation of Toll-like and NOD-like receptor (TLR/NLR) signaling, lipid and atherosclerosis pathways, neutrophil extracellular trap (NET) formation, complement system, NF-κB, and MAPK signaling (Figure 3A–C). These findings suggest immune dysregulation as a prominent characteristic of APS pathogenesis.

The prominent activation of TLR and other PRR pathways prompted us to further explore their role in APS pathogenesis. PRRs recognize pathogen- and damage-associated molecular patterns to initiate immune responses [38]. Their expression on platelets suggests a potential mechanistic interface between platelet activation and inflammatory signaling. In APS, activated platelets release mediators that recruit neutrophils, which subsequently generate NETs that further stimulate platelet activation, thereby establishing a self-amplifying loop of immunothrombosis [39,40]. To further delineate these pathways, we visualized key genes involved in PRR signaling and neutrophil chemotaxis. The coordinated upregulation of these genes in APS compared with carriers is consistent with close transcriptional coupling between platelets and innate immune–neutrophil networks (Figure 3D).

We also applied machine learning to distinguish APS from aPL carriers and identified six key genes—*CMTM2*, *COPS4*, *FCER1G*, *POLR2G*, *SACM1L*, *VBP1*—consistently selected by at least two algorithms (Figure 3E,F), with discriminative performance ranging from AUC 0.79 to 0.85 (Figure 3G). The detailed machine learning process, including gene selection by three algorithms, is illustrated in Supplementary Figure S5.

### 2.5. Antibody-Specific Transcriptional Reprogramming of Platelets in APS

**Figure 4:**
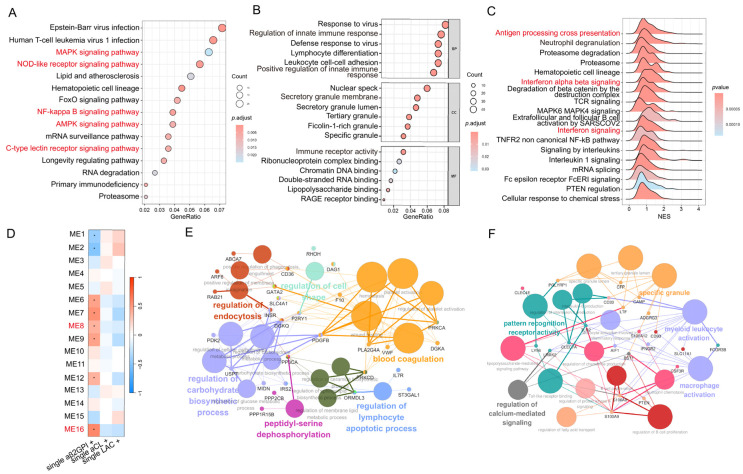
Impact of antiphospholipid antibody titers and types on platelet transcriptional profiles. (**A**–**C**) Comparisons between high-titer aPL-positive APS patients and healthy controls. (**A**) Dot plot showing KEGG analysis. (**B**) Dot plot showing GO analysis. (**C**) Ridge plot showing the GSEA of canonical pathways. (**D**) Heatmap showing gene modules derived from WGCNA using platelet transcriptomic data of APS patients with single aβ2GPI positive, single aCL positive and single LA positive. Asterisks indicate statistically significant correlations between modules (rows) and aPLs (columns). (**E**) GO Pathway enrichment network of module 16 identified by WGCNA, reflecting the pathologic effect of aβ2GPI from a pro-coagulation perspective. (**F**) GO Pathway enrichment network of module 8 identified by WGCNA, reflecting the pathologic effect of aβ2GPI from an immune activation perspective. GO pathway enrichment network was generated by ClueGO software in Cytoscape. Large nodes represent enriched GO pathways, with node size proportional to the significance of enrichment. Small nodes depict individual genes shared between pathways, illustrating the functional relationships among the enriched pathways. WGCNA, weighted gene coexpression network analysis; aPL, antiphospholipid antibody; APS, antiphospholipid syndrome; aβ2GPI, anti-β2-glycoprotein 1; aCL, anti-cardiolipin; LAC, lupus anticoagulant. * *p* < 0.05. Red-colored elements in this figure are those specifically highlighted and discussed in the main text.

High-titer and triple-positive aPLs, which define a high-risk profile and are associated with increased thrombotic risk, are considered as the “first hit” in the “two-hit model” of thrombosis [41]. Platelets from APS patients show markedly enhanced activation in vitro, and aPL-”educated” platelets—considered the main responders to the “second hit” [42]—are thought to reflect the pathogenic effects of aPLs at the transcriptional level. Functional enrichment analysis of DEGs in high-titer or triple-positive APS versus healthy controls revealed up-regulation of AMPK, MAPK, and NF-κB signaling pathway-mediated platelet activation. More importantly, enrichment of PRR pathways, interferon signaling, and antigen processing and cross-presentation suggests heightened immune-related transcriptional activity in the presence of high-risk aPLs (Figure 4A–C, Supplementary Figure S6).

To dissect the effects of different aPL types on platelet transcription, we performed WGCNA on patients positive for single aCL, aβ2GPI, or LAC, identifying 16 co-expression gene modules (Figure 4D). Six modules were significantly associated with aβ2GPI positivity, with modules 8 and 16 selected for in-depth analysis based on functional enrichment and significance. Module 16 was enriched in blood coagulation, membrane lipid metabolism, endocytosis, cytoskeletal reorganization, carbohydrate metabolism, and lymphocyte apoptosis, with key genes including *CD36*, *GATA2*, *PLA2G4A*, *VWF*, *PRKCD*, *ORMDL3*, *PPP3CA*, and *USP7*, suggesting significant transcriptional link between aβ2GPI positivity and platelet activation and coagulation (Figure 4E). Module 8 was enriched in myeloid leukocyte and macrophage activation, vesicle formation, PRR activity, and calcium signaling, with key genes such as *LTF*, *CLEC7A*, *S100A8*, *S100A9*, *PTEN*, *S100A12*, *IFNGR2*, *SLC11A1*, and *FCGR3B*, suggesting aβ2GPI-associated transcriptional reprogramming toward immunomodulatory functions in platelets (Figure 4F).

Overall, we found platelets from high-titer and triple-positive aPL carriers exhibit enhanced immune-related transcriptional profiles, while WGCNA identifies gene co-expression modules associated with aβ2GPI positivity that are enriched in both coagulation and immunoregulatory pathways.

### 2.6. Distinct Transcriptomic Signatures and Mechanisms in Arterial vs. Venous Thrombosis

**Figure 5:**
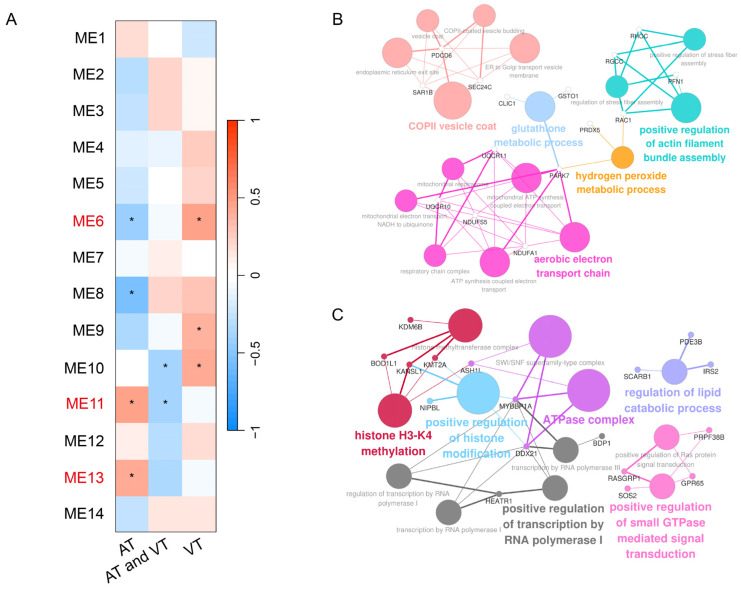
Unsupervised cluster analysis reveals distinct pathogenetic mechanisms in APS-related venous and arterial thrombosis. (**A**) Heatmap showing gene modules derived from WGCNA using platelet transcriptomic data of APS patients with arterial thrombosis, venous thrombosis, or both. Asterisks indicate statistically significant correlations between modules (rows) and APS-related clinical manifestations (columns). (**B**) GO Pathway enrichment network of module 11 and 13 identified by WGCNA, reflecting transcriptional changes in arterial thrombosis platelets associated with APS. (**C**) GO Pathway enrichment network of module 6 identified by WGCNA, reflecting transcriptional changes in venous thrombosis platelets associated with APS. GO pathway enrichment network was generated by ClueGO and CluePedia software in Cytoscape. Large nodes represent enriched GO pathways, with node size proportional to the significance of enrichment. Small nodes depict individual genes shared between pathways, illustrating the functional relationships among the enriched pathways. WGCNA, weighted gene coexpression network analysis; APS, antiphospholipid syndrome; AT, arterial thrombosis; VT, venous thrombosis. * *p* < 0.05. Red-colored elements in this figure are those specifically highlighted and discussed in the main text.

Venous and arterial thrombosis are distinct entities that differ in pathogenesis, clinical manifestations, and therapeutic approaches. Arterial thrombosis is predominantly driven by platelet activation and aggregation, whereas venous thrombosis is mainly mediated by activation of the coagulation cascade, with platelets playing a secondary role [43,44]. To explore transcriptomic differences underlying these thrombotic phenotypes in APS, we performed WGCNA and identified co-expression gene modules associated with arterial and venous events. Four modules were positively correlated with APS-related arterial thrombosis, while three modules were linked to venous thrombosis (Figure 5A).

Modules associated with arterial thrombosis were enriched in pathways related to protein secretion, cytoskeletal organization, and mitochondrial function. Representative hub genes included small GTPases (*RHOC*, *RAC1*), the actin-binding protein *PFN1*, and mitochondrial respiratory chain components (*PARK7*, *UQCR10*, *NDUFA1*, *NDUFS5*). The enrichment of pathways is consistent with the high energy demand and cytoskeletal remodeling required for platelet activation and aggregation (Figure 5B). In contrast, modules correlated with venous thrombosis were enriched in pathways involving small GTPase-mediated signaling, lipid metabolism, and transcriptional regulation. Key genes included chromatin modifiers (*KANSL*, *KMT2A*) and guanine nucleotide exchange factors (*RASGRP1*), suggesting their roles in intracellular signaling and gene expression (Figure 5C).

While these transcriptomic distinctions are consistent with known pathogenic differences, their specificity to arterial versus venous thrombosis in APS warrants further investigation. Future studies integrating functional validation with multi-omics approaches will be essential to establish causal mechanisms and clarify the differential contributions of these transcriptional programs to arterial versus venous thrombosis in APS.

## 3. Discussion

Our study delineates the platelet transcriptomic landscape across the spectrum of aPL positivity, from asymptomatic carriers to APS patients. These findings build upon accumulating evidence that platelets contribute not only to thrombosis but also to immune dysregulation in APS [2,3,4,5]. By integrating differential expression, pathway analysis, WGCNA, and machine learning, we define a distinct, pro-thrombotic, and immunologically active platelet phenotype unique to APS. Our findings position the platelet not merely as a passive target but as an active participant in APS pathogenesis, offering a framework for diagnostic stratification and mechanism-based heterogeneity.

Our data suggest that the platelet transcriptome serves as a sensitive molecular sensor distinguishing APS patients from both healthy individuals and asymptomatic aPL carriers. This molecular divergence underscores a fundamental reprogramming of platelet biology in the transition from carrier state to clinical disease. The activated pathways—notably NF-κB, MAPK, and AMPK signaling—indicate enhanced platelet activation and immune regulation, consistent with thrombo-inflammation [45]. Critically, this transcriptional signature is not a mere epiphenomenon of platelet turnover, as its overlap with the young, reticulated platelet transcriptome was minimal (<6%). This suggests the reprogramming is stable and disease-intrinsic, not age-related.

Central to this APS-specific phenotype is the marked enrichment of innate immune sensing pathways, particularly TLR signaling, together with activation of NLR-associated transcriptional programs. Their activation suggests aPLs may act as endogenous ligands engaging these receptors, shifting platelets toward an immunologic role. While TLR activation by anti-β2GPI antibodies has been reported in other cell types [46,47,48], our transcriptomic analysis extends these observations to the platelet compartment—to our knowledge, providing the first systematic characterization of TLR/NLR pathway reprogramming in APS platelets. Our findings are consistent with a model in which autoantibodies contribute to sustained platelet inflammation. Downstream of PRR engagement, robust activation of NF-κB and MAPK cascades likely amplifies pro-thrombotic and pro-inflammatory mediator production, potentially creating a self-reinforcing circuit. This mechanistic interpretation is supported by recent evidence demonstrating that aPLs directly activate platelets through ERK/p38 MAPK signaling and that lipid raft-associated signaling platforms contribute to aPL-mediated cellular activation [49,50]. Consistent with this membrane-associated signaling reprogramming, we observed differential regulation of established aPL-binding receptors: upregulation of activating receptor *FCGR2A* (FcγRIIa, log_2_FC = 0.81) and downregulation of *LRP8* (ApoER2, log_2_FC = −1.48). This reciprocal pattern is consistent with a shift toward enhanced platelet reactivity and altered regulatory signaling.

Notably, this PRR-driven signature co-exists with transcriptional programs related to neutrophil chemotaxis and NET formation. Coordinated gene upregulation suggests potential functional interplay between activated platelets and neutrophils in APS. We propose a testable model wherein PRR-stimulated platelets release cytokines and chemokines that recruit and prime neutrophils, which in turn release NETs that further activate platelets and promote thrombosis. This putative “platelet-neutrophil feedback loop” is consistent with established immunothrombosis paradigms [39] and offers a potential pathophysiological explanation for potentiated thrombotic risk in APS. Our data provide the transcriptional groundwork for this model, highlighting it as a priority for future functional validation.

This immunothrombotic phenotype has two translational implications. First, our results show that machine learning can distill this complex platelet transcriptome into a compact gene signature with high discriminative capacity (AUC 0.901–0.948). This suggests platelet RNA-derived signatures, particularly the 15-gene panel identified by our machine learning pipeline, could serve as novel biomarkers for distinguishing APS patients from healthy controls, complementing current serological and clinical criteria, although external validation will be required before clinical application. Importantly, the ability to distinguish APS from asymptomatic aPL carriers may provide insight into molecular changes associated with progression from serological autoimmunity to overt clinical disease. Key genes within this signature, such as *FCER1G* (an immunoreceptor adaptor) and *MAP4K4* (a proinflammatory kinase), are themselves nodes within the broader immune activation networks we identified, potentially linking diagnostic potential to pathogenesis. Second, our network analyses identified previously unappreciated heterogeneity within APS. The transcriptomic impact of aPLs is not uniform; anti-β2GPI antibodies associate with the most robust co-expression modules, tying directly to both coagulation (e.g., *VWF*) and immune effector (e.g., *S100A8/A9*) genes. This may explain the particularly high thrombotic risk linked to this antibody specificity [51]. Furthermore, we identified distinct transcriptional programs associated with arterial versus venous thrombotic events. The arterial signature, enriched for cytoskeletal and mitochondrial pathways, is consistent with the biomechanical demands of platelet aggregation under high shear stress. In contrast, the venous signature points toward lipid metabolism and transcriptional regulation, suggesting a pathophysiology with different emphases. This molecular stratification lays the groundwork for moving beyond a one-size-fits-all view of APS toward more personalized risk assessment.

Our study provides a foundational transcriptomic map of APS platelets, with findings both supported by and extending beyond existing mechanistic knowledge. While our cohort enabled robust discovery, validation in larger cohorts and extension beyond the classical aPL triad (e.g., anti-prothrombin antibodies [52]) are warranted. Several additional limitations should be acknowledged. First, all participants were recruited from a single center in Shanghai; validation in multi-ethnic cohorts is therefore needed. Second, although all APS patients were newly diagnosed, treatment-naïve, and had a relatively short disease duration at enrollment, clinical heterogeneity within APS may contribute to platelet transcriptomic variability. Future studies using larger, clinically balanced cohorts with detailed longitudinal thrombotic histories will be necessary to clarify how clinical heterogeneity—including differences in thrombotic burden (e.g., single versus recurrent thrombotic events)—influences platelet transcriptomic profiles. Third, platelets can take up exogenous RNAs from circulation [53], raising the possibility that some transcriptomic signatures may reflect passive RNA uptake rather than intrinsic platelet reprogramming. Nevertheless, our stringent isolation protocol minimized cellular contamination, and the coordinated enrichment of disease-relevant pathways argues against passive RNA acquisition as the primary explanation for our findings.

Despite these limitations, our transcriptional data generate a specific, testable hypothesis: that TLR/PRR-driven platelet activation underpins enhanced platelet–neutrophil collaboration in APS. While prior functional studies have established that APS promotes neutrophil–platelet aggregate formation [54], our work identifies upstream transcriptional reprogramming in platelets that may predispose them to such interactions. Direct experimental validation is now needed to causally link our transcriptional signature to this established functional phenotype.

In conclusion, our multi-modal transcriptomic analysis suggests that platelets in APS acquire a distinct proinflammatory phenotype driven by innate immune recognition pathways. This biological insight may inform future diagnostic strategies and support a novel, testable mechanism for disease propagation involving neutrophil recruitment. By elucidating antibody-specific and thrombosis-type-specific molecular subnetworks, this work provides a roadmap for developing precision diagnostics and targeting key drivers of immunothrombosis in APS.

## 4. Materials and Methods

### 4.1. Experimental Design

We enrolled patients with APS and asymptomatic aPL carriers, recruited from the APS-SH (Antiphospholipid Syndrome-Shanghai) cohort at Ruijin Hospital, Shanghai. For comparison, healthy controls matched by age and sex were also enrolled. APS patients were classified according to the 2023 ACR/EULAR classification criteria [1]. Lupus anticoagulant (LAC) was detected according to International Society of Thrombosis and Hemostasis (ISTH) recommendations [55], employing a standardized three-step process involving screening, mixing, and confirmatory assays. Levels of aCL and anti-β2GPI antibodies (both IgG and IgM isotypes) were quantified via enzyme-linked immunosorbent assay (ELISA). Based on the 2023 ACR/EULAR criteria, aPL profiles were designated as moderate- or high-titer, with cut-offs of ≥40 units and ≥80 units for aCL and anti-β2GPI antibodies, respectively [1].

### 4.2. Platelet Isolation and mRNA Sequencing

Whole blood samples were collected from the antecubital fossa using buffered sodium citrate tubes. Following isolation via two sequential centrifugation steps, platelets were resuspended in RNAlater (Thermo Scientific, Waltham, MA, USA). Total RNA was purified using the RNeasy Micro Kit (Ambion; Thermo Scientific; catalog #AM156), and its integrity was subsequently evaluated with a Qubit 2.0 Fluorometer and an RNA 6000 Pico chip on a Bioanalyzer 2100 system (Agilent, Santa Clara, CA, USA). cDNA library construction and mRNA sequencing followed procedures from a previous study [56]. Leukocyte contamination was <0.1% by flow cytometry, with nearly undetectable CD45 RNA by qRT-PCR.

### 4.3. Sequencing Data Processing

FASTP (v0.23.2) was used to filter raw fastq data by removing adapters, PCR primers, and low-quality reads. After alignment of clean data to the human Ensembl 91 genome with STAR (v2.4.2a), the generated BAM files were subjected to gene-level quantification via HTSeq (v0.6.1). Quality control involved filtering the initially quantified genes by removing those with (i) fewer than ten reads in over 50% of samples, (ii) zero counts in over half of the samples, and (iii) all ribosomal protein-coding genes, resulting in a final set of 18,357 genes for analysis.

### 4.4. Differential Expression Analysis

Data normalization employed the voom function in the limma package (v3.58.1). Differential expression analysis was performed using empirical Bayes moderated linear models. For comparisons across three groups, an overall F-test was conducted followed by pairwise contrasts. Differential expression analysis via the limma pipeline identified DEGs across multiple comparisons: (1) aPL-positive individuals versus HC; (2) APS patients versus HC; (3) asymptomatic aPL carriers versus HC; (4) APS patients versus asymptomatic aPL carriers; (5) high-titer aPL APS patients versus HC; (6) triple positive aPL APS patients versus HC. Genes characterized by |log_2_FC| ≥ 1 and a *p*-value < 0.05 were considered differentially expressed. To interpret their biological functions, enrichment analysis of KEGG and GO pathways was performed using the clusterProfiler package (v4.10.1). A dot plot was generated to visualize top enrichment pathways with pathways ordered by gene ratio and colored by *p*-adjust.

### 4.5. Gene Set Enrichment Analysis

GSEA was conducted using clusterProfiler package (v4.10.1) with the Molecular Signatures Database’s Canonical Pathways gene sets (c2.cp.v2024.1.Hs.symbols). A ranked gene list was constructed based on the product of −log_10_(*p*-value) and log_2_FC obtained from the limma analysis. Significant enrichment was determined by the normalized enrichment score (NES) and a False Discovery Rate (FDR)-adjusted *p*-value threshold of less than 0.05. A ridge plot was generated to visualize top enrichment pathways, with pathways ordered by NES and colored by *p*-value.

### 4.6. Weighted Gene Co-Expression Network Analysis

An unsupervised weighted gene co-expression network was built using the WGCNA package (v1.73) to define gene modules. Modules reaching statistical significance (*p* < 0.05) were annotated for functional enrichment via clusterProfiler, revealing their biological themes. To pinpoint disease-relevant signatures, the identified modules were subsequently examined for correlations with both clinical manifestations and autoantibody status.

### 4.7. Protein–Protein Interaction Networks and Pathway–Gene Complex Network

PPI network construction was performed in the STRING platform (v12.0), inputting genes from WGCNA modules (arterial: module 11 and 13; venous thrombosis: module 6; anti-β2GPI antibody positive: module 8 and 16). Settings were restricted to high-confidence (*Homo sapiens*, physical interactions, confidence score > 0.5). The high-confidence interaction networks were downloaded as TSV files for downstream analysis and visualization in Cytoscape (v3.10.2). Additionally, a pathway–gene complex network was constructed to illustrate the relationships between key pathways and their associated genes. To discover the functional landscape, module-based GO and pathway enrichment was executed via ClueGO and CluePedia software (v2.5.10 and v1.5.10, respectively) in Cytoscape. Terms with statistical significance (*p* < 0.05) were analyzed to uncover the molecular interplay involved in APS-related thrombosis and immune modulation.

### 4.8. Machine Learning-Based Classification and Feature Selection

To distinguish APS patients from HC and APS patients from asymptomatic aPL carriers, three distinct machine learning approaches were used: LASSO regression, RF, and SVM-RFE for feature gene selection. LASSO regression, characterized by fitting generalized linear models with variable selection, was conducted via the glmnet package (v4.1.8). The optimal regularization parameter (λ) was determined through 10-fold cross-validation. Using the randomForest package (v4.7.1.2), RF was applied to assess gene importance, with ranking based on the mean decrease in the Gini index. SVM-RFE, based on the maximum margin principle of SVM, minimizes structural risk and empirical error to enhance model performance and was implemented using the e1071 package (v1.7.16). Genes consistently identified by at least two of the three methods were selected as key discriminative features. The potential classification utility of these candidate biomarkers was subsequently assessed by plotting ROC curves and calculating the corresponding AUC.

### 4.9. Statistical Analysis

Statistical analyses were performed using R software (v4.3.1). Unless otherwise noted, a two-tailed *p* < 0.05 was considered significant, with multiple-comparison adjustment via the Benjamini–Hochberg method where appropriate.

## Figures and Tables

**Table 1:** Characteristics of patients with APS, aPL carriers and healthy control included in the study.

	APS	aPL Carrier	HC
N	43	20	20
Female, *n* (%)	28 (65.1%)	19 (95%)	12 (60%)
Age, Mean ± SD	38.9 ± 12.8	36.5 ± 9.1	43 ± 16.8
Clinical characteristics			
Primary APS, *n* (%)	30 (69.8%)	/	/
Secondary APS, *n* (%)	13 (30.2%)	/	/
Venous thromboembolism, *n* (%)	25 (58.1%)	0 (0%)	0 (0%)
Arterial thrombosis, *n* (%)	21 (48.8%)	0 (0%)	0 (0%)
Microvascular thrombosis, *n* (%)	4 (9.3%)	1 (5%)	0 (0%)
Obstetric morbidity, *n* (%)	8 (18.6%)	0 (0%)	0 (0%)
Cardiac valve disease, *n* (%)	4 (9.3%)	0 (0%)	0 (0%)
Thrombocytopenia *, *n* (%)	11 (25.6%)	4 (20%)	0 (0%)
Laboratory characteristics			
LAC positive, *n* (%)	39 (90.7%)	14 (70%)	0 (0%)
Anti-CL positive, *n* (%)	23 (53.5%)	17 (85%)	0 (0%)
Moderate positive, *n* (%)	4 (9.3%)	6 (30%)	0 (0%)
High positive, *n* (%)	19 (44.2%)	11 (55%)	0 (0%)
Anti-β2GPI positive, *n* (%)	21 (48.8%)	15 (75%)	0 (0%)
Moderate positive, *n* (%)	10 (23.2%)	4 (20%)	0 (0%)
High positive, *n* (%)	11 (25.6%)	11 (55%)	0 (0%)
Triple positive, *n* (%)	17 (39.5%)	11 (55%)	0 (0%)

APS, antiphospholipid syndrome; aPL, antiphospholipid antibody; HC, healthy control; LAC, lupus anticoagulant; anti-CL, anti-cardiolipin; anti-β2GPI, anti-β2-glycoprotein 1. * The threshold of thrombocytopenia was established at 100 × 10^9^/L.

## Data Availability

All data are available in the main text or the Supplementary Materials. Further information and requests for resources and reagents should be directed to and will be fulfilled by the Lead Contact, Tingting Liu, at liutingting9905@163.com. The raw sequence data reported in this paper have been deposited in the Genome Sequence Archive in National Genomics Data Center [57], China National Center for Bioinformation/Beijing Institute of Genomics, Chinese Academy of Sciences (GSA-Human: HRA014007) that will be publicly accessible at https://ngdc.cncb.ac.cn/gsa-human after 19 October 2027.

## Supplementary Materials

The following supporting information can be downloaded at: https://www.mdpi.com/article/10.3390/ijms27125428/s1.

## Abbreviations

The following abbreviations are used in this manuscript:

APS	Antiphospholipid Syndrome
aβ2GPI/anti-β2GPI	anti-β2-glycoprotein I antibody
aCL	anticardiolipin antibodies
aPL(s)	antiphospholipid antibody(ies)
AUC	Area Under the Curve
DEG(s)	Differentially Expressed Gene(s)
ELISA	Enzyme-Linked Immunosorbent Assay
FDR	False Discovery Rate
GO	Gene Ontology
GSEA	Gene Set Enrichment Analysis
HC(s)	Healthy Control(s)
ISTH	International Society on Thrombosis and Haemostasis
KEGG	Kyoto Encyclopedia of Genes and Genomes
LAC	Lupus Anticoagulant
LASSO	Least Absolute Shrinkage and Selection Operator
LDA	Linear Discriminant Analysis
log_2_FC	log_2_ Fold Change
MAPK	Mitogen-Activated Protein Kinase
NET(s)	Neutrophil Extracellular Trap(s)
NF-κB	Nuclear Factor Kappa B
NLR	NOD-Like Receptor
PPI	Protein–Protein Interaction
PRR(s)	Pattern Recognition Receptor(s)
RF	Random Forest
ROC	Receiver Operating Characteristic
SLE	Systemic Lupus Erythematosus
SVM-RFE	Support Vector Machine-Recursive Feature Elimination
TLR	Toll-Like Receptor
WGCNA	Weighted Gene Co-expression Network Analysis
