# Supplementary material for: Deciphering Platelet Transcriptomic Profiles in Antiphospholipid Syndrome: Insights into Their Role as Immunological Players
Source: Int J Mol Sci. 2026 Jun 16;27(12):5428. doi: 10.3390/ijms27125428 (PMC13300163; doi:10.3390/ijms27125428)
Supplement: Supplementary file 1 [file ijms-27-05428-s001.zip › ijms-4339587-supplementary.pdf]

Supplementary Materials

Deciphering Platelet Transcriptomic Profiles in Antiphospholipid Syndrome: Insights into Their Role as Immunological Players

Note: Red-colored elements in figures indicate pathways or modules that are specifically highlighted and discussed in the main text.

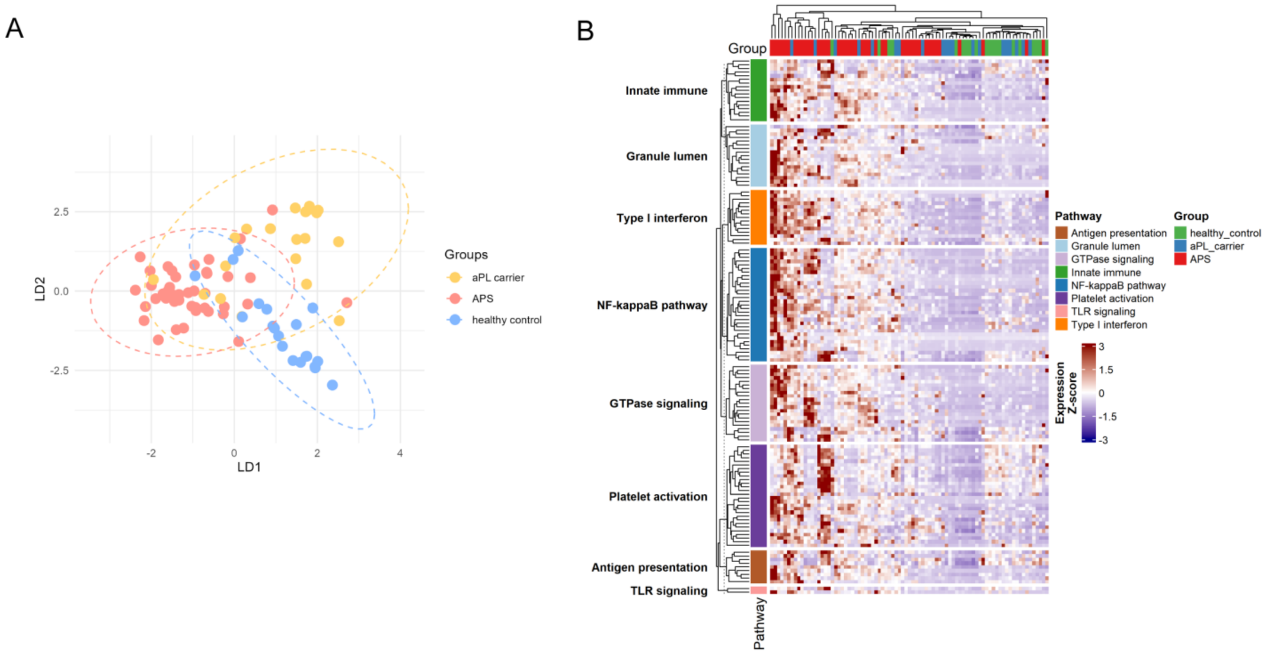

Supplementary Figure S1. Transcriptomic separation and pathway expression across APS, aPL carriers, and healthy controls. (**A**) LDA plot derived from the global platelet transcriptome demonstrating distinct clustering of samples from APS patients (N = 43), aPL carriers (N = 20) and HC (N = 20). (**B**) Heatmap depicting the normalized expression (Z-score) of genes comprising the top significantly enriched pathways identified from the comparison of APS, aPL carriers and HC. Rows are grouped into modules corresponding to these enriched pathways, with genes within each module shown. Columns represent individual samples, which were hierarchically clustered based on their expression profiles across these genes. Sample groups are annotated above the heatmap. LDA, Linear Discriminant analysis; APS, antiphospholipid syndrome; aPL, antiphospholipid antibody; HC, healthy controls.

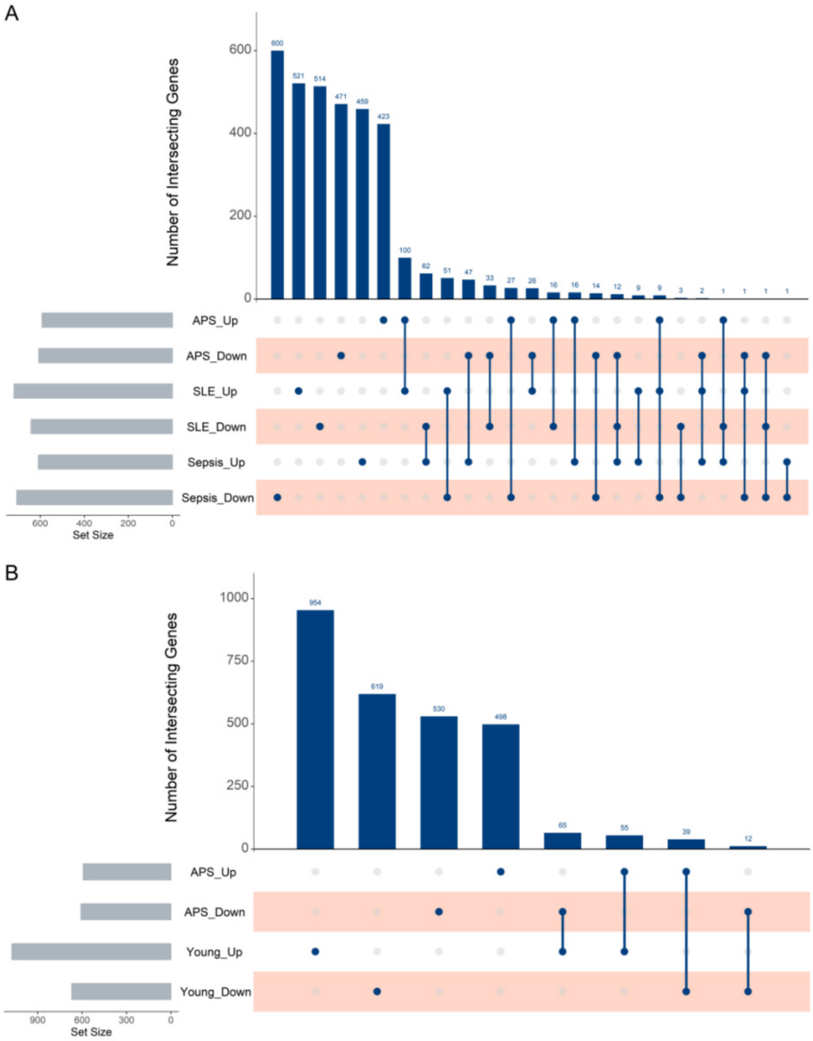

Supplementary Figure S2. Specificity of the APS platelet transcriptomic signature assessed by cross-condition comparison. (**A**) Comparison with SLE and sepsis. UpSet plot showing intersections among DEG sets (up- and down-regulated) from APS, SLE, and sepsis platelets. Bar height indicates intersection size. (**B**) Comparison with reticulated platelets. UpSet plot showing intersections between APS DEG sets and a published reticulated (young) platelet transcriptome. Minimal overlap indicates the APS signature is independent of platelet age. APS, antiphospholipid syndrome; SLE, systemic lupus erythematosus; DEGs, differentially expressed genes.

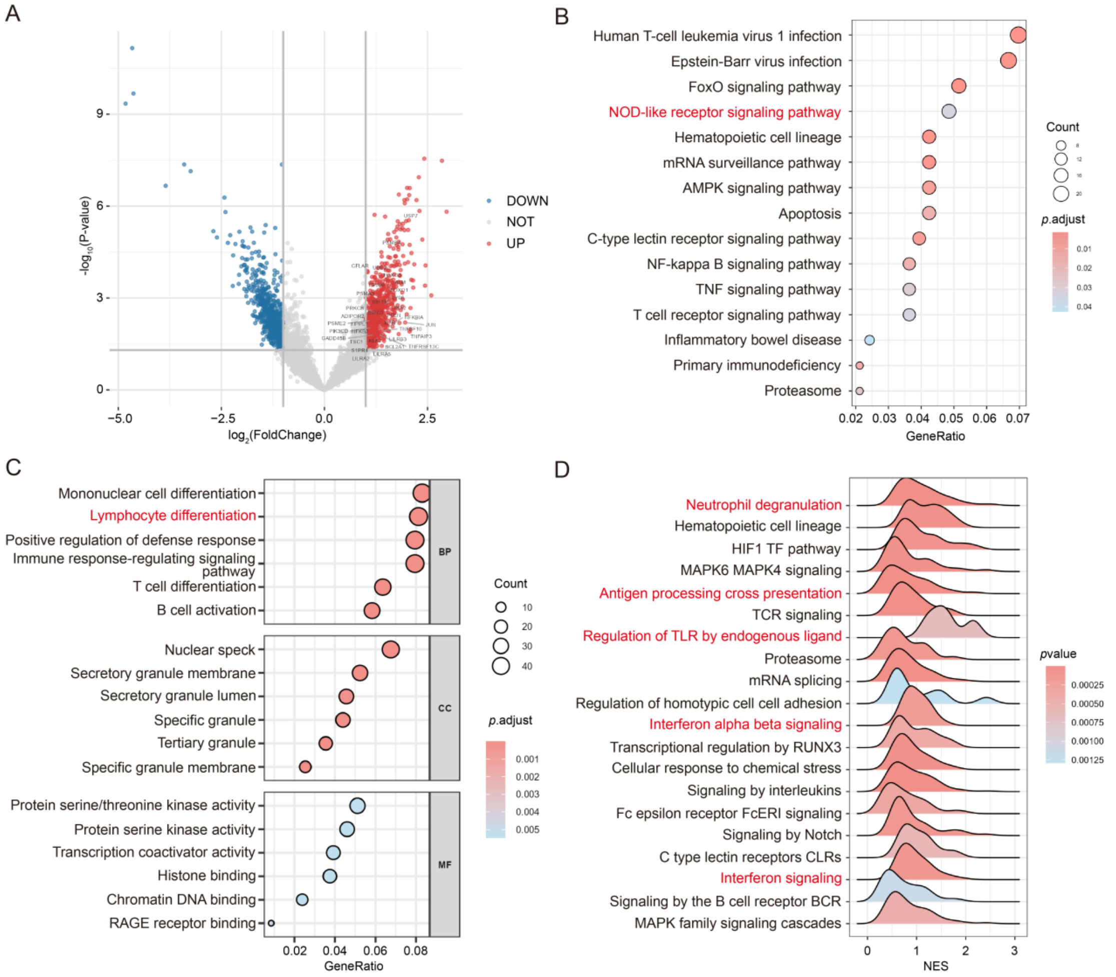

Supplementary Figure S3. Platelet transcriptomic signature in APS patients versus healthy individuals. (**A**) Volcano plot showing DEGs. (**B**) Dot plot showing KEGG analysis. (**C**) Dot plot showing GO analysis. (**D**) Ridge plot showing the GSEA of canonical pathways. DEGs, differentially expressed genes; APS, antiphospholipid syndrome; HC, healthy controls; KEGG, Kyoto Encyclopedia of Genes and Genomes; GO, gene ontology; GSEA, gene set enrichment analysis; NES, normalized enrichment score.

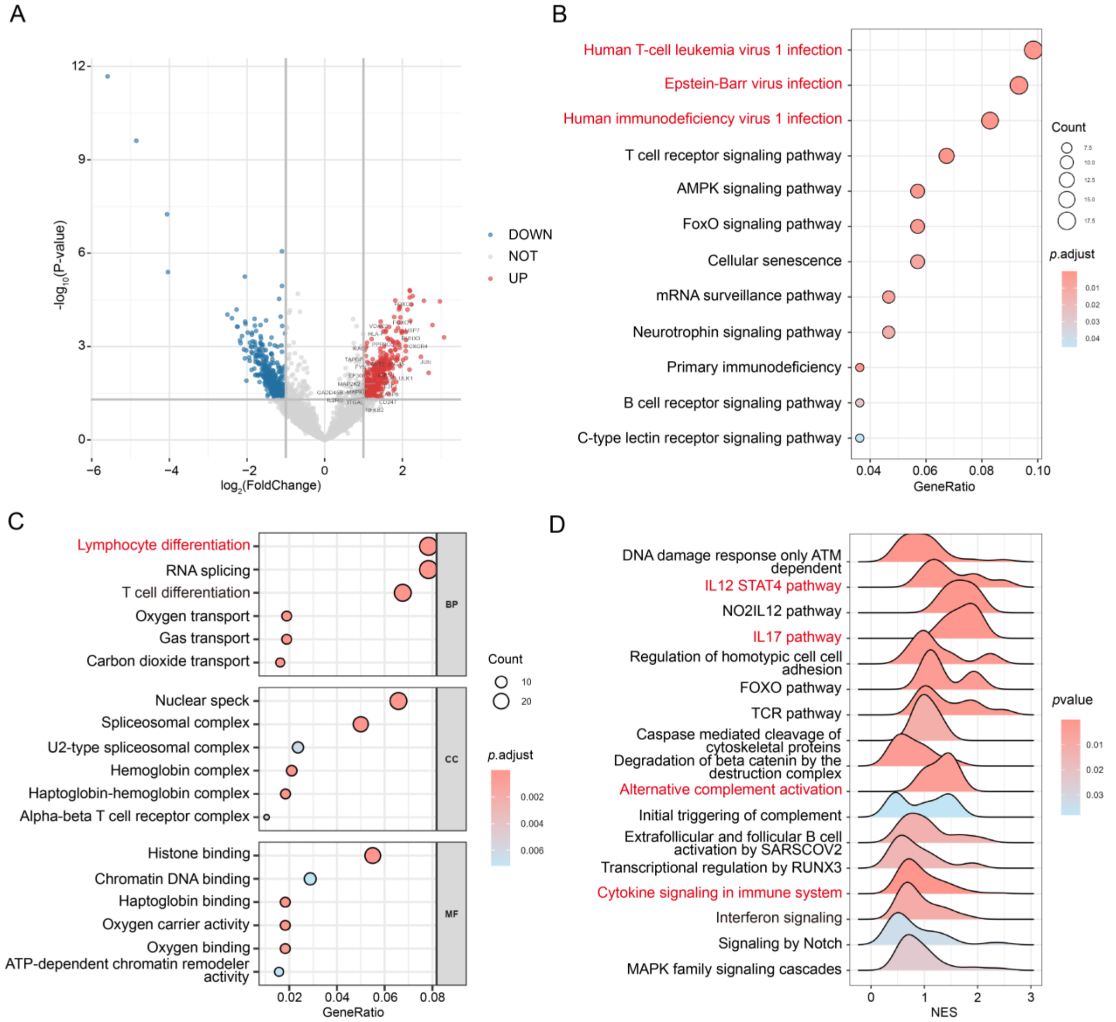

Supplementary Figure S4. Platelet transcriptomic signature in aPL carriers versus healthy individuals. (**A**) Volcano plot showing DEGs. (**B**) Dot plot showing KEGG analysis. (**C**) Dot plot showing GO analysis. (**D**) Ridge plot showing the GSEA of canonical pathways. DEGs, differentially expressed genes; aPL, antiphospholipid antibody; HC, healthy controls; KEGG, Kyoto Encyclopedia of Genes and Genomes; GO, gene ontology; GSEA, gene set enrichment analysis; NES, normalized enrichment score.

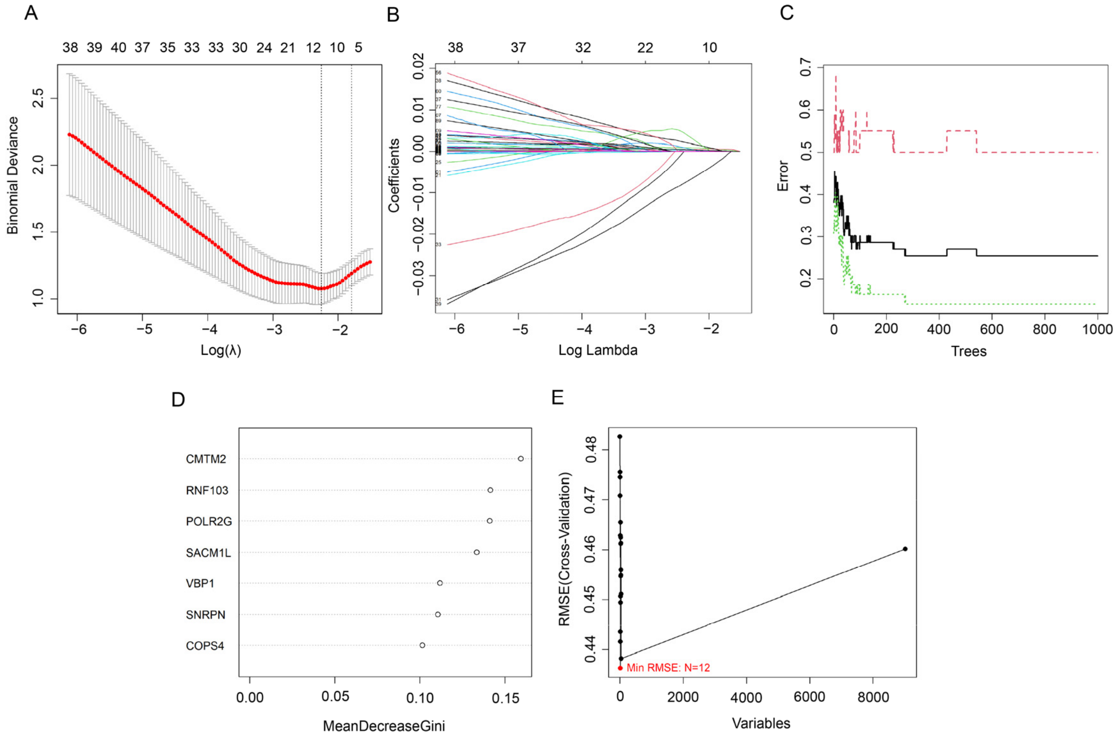

Supplementary Figure S5. Machine learning identifies key discriminative genes of APS versus aPL carriers. (**A**,**B**) The performance of 10-fold cross verification for tuning parameter in selection LASSO. (**C**) Random forest error rate versus the number of classification trees. (**D**) Top 7 relatively important genes selected by randomForest. (**E**) Recursive feature elimination (SVM–RFE) algorithm selection.

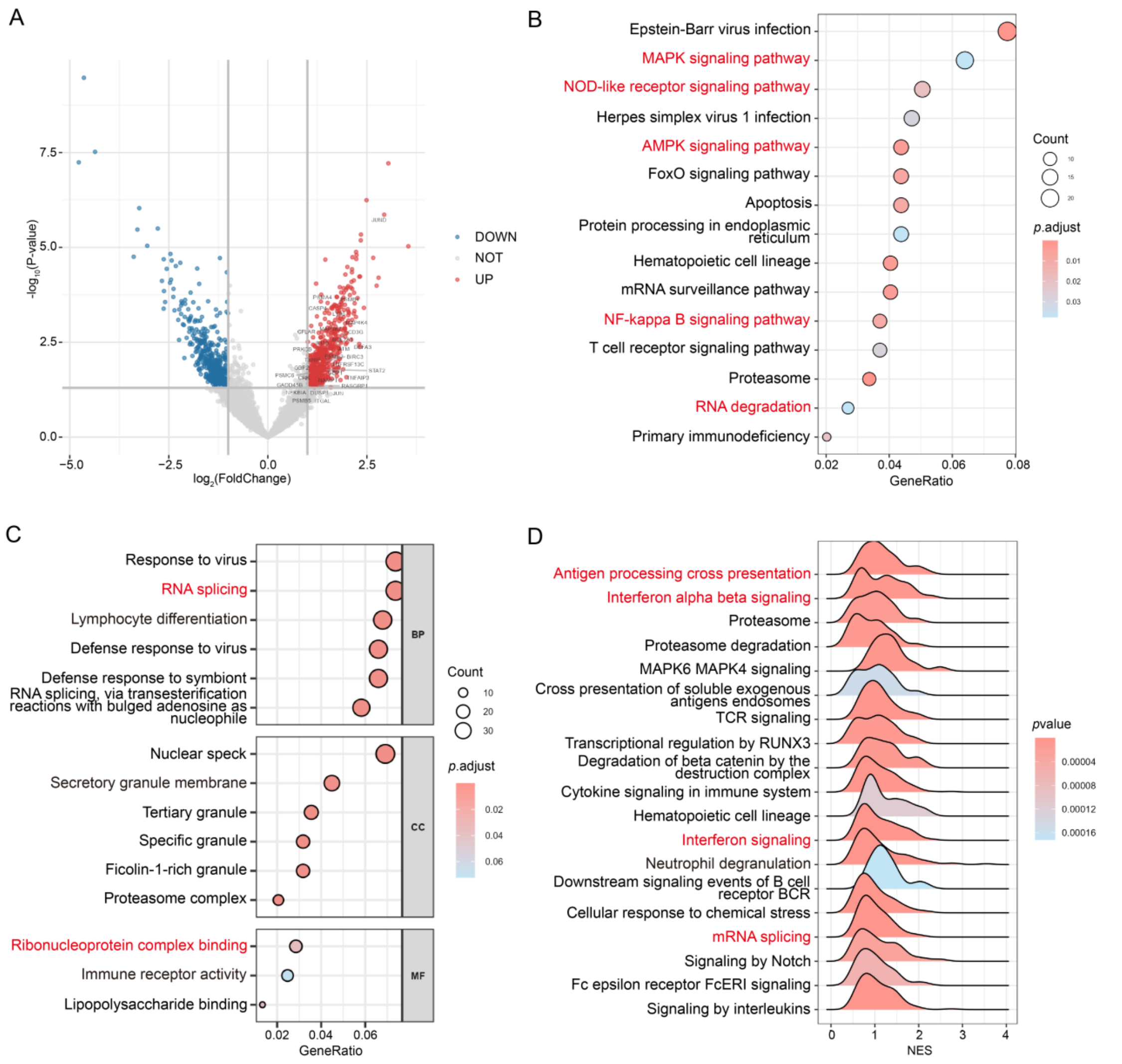

Supplementary Figure S6. Platelet transcriptomic signature in triple positive APS patients versus healthy individuals. (**A**) Volcano plot showing DEGs. (**B**) Dot plot showing KEGG analysis. (**C**) Dot plot showing GO analysis. (**D**) Ridge plot showing the GSEA of canonical pathways. DEGs, differentially expressed genes; HC, healthy controls; KEGG, Kyoto Encyclopedia of Genes and Genomes; GO, gene ontology; GSEA, gene set enrichment analysis; NES, normalized enrichment score.
